# On the need for a sexual healthcare commissioner

**DOI:** 10.1136/sextrans-2024-056223

**Published:** 2024-05-23

**Authors:** George J Severs

**Affiliations:** International History and Politics; Gender Centre, Graduate Institute of International and Development Studies, Geneva, Switzerland

**Keywords:** HIV, SEXUAL HEALTH, POLICY

A recent *Guardian* analysis found that English councils have cut spending on sexual health services by more than one-third since 2013, coinciding with a rise in hospitalisation for sexual health issues.^1^ These findings suggest an alarming effect of austerity measures, but this is not the whole picture. Lack of holistic commissioning oversight has also played a part, especially for services which require both sexual health *and* HIV care. Here, I argue that a commissioner for sexual health services is needed.

Sexual assault and rape cases make this lack of holistic commissioning stark. In May 2022, I convened a policy-focused roundtable which examined the state of HIV and sexual healthcare available following acts of sexual violence in England.^2^ The barriers it identified to accessing post-rape care and services highlighted wider issues facing sexual health clinicians, issues which remain relevant two years on as they continue to adapt to the introduction of Integrated Care Systems (ICS). Two clear policy recommendations emerged. First, sexual health services needed urgently to be brought back under the National Health Service’s (NHS) commissioning scope. Second, a national commissioner for sexual health should be appointed with oversight of sexual health services across England.

Clinical experts and service providers identified a lack of top-down oversight as a major cause of uneven post-rape care. A leading forensic physician suggested that ‘one of the issues for sexual violence [healthcare] is that no one organisation takes overall responsibility… no one’s looking at it in the round and from a patient’s, victim’s, complainant’s perspective for the whole journey’. A sexual healthcare commissioner was identified as a means of overseeing the care of patients who required both HIV and sexual health services holistically. This was compared with the field of domestic violence, where the domestic abuse commissioner has oversight. In other national contexts, dedicated commissioners advise legislators on matters relating to HIV and sexual health, such as the Federal Commission for Issues relating to Sexually Transmitted Infections in Switzerland.

Speaking before the introduction of ICS, the medics argued that sexual health services should be commissioned under the NHS, like HIV care. Despite the attempt at greater integration under ICS since 2022, a division of commissioning remains between HIV and sexual health services.^3^ One HIV consultant articulated the impact that the split in commissioning was having on services (such as post-sexual assault care) where both HIV *and* sexual healthcare are at play. For her, the impact was felt in the loss of health advisors: ‘we used to have health advisors but we only have them on our HIV side now… For the sexual health side of our service they don’t have that any more’.

This loss of health advisors within clinics speaks to two major challenges facing clinicians: lack of resource and lack of capacity. Austerity and split commissioning exacerbated this lack of resource. In the words of a forensic sexual health medic, ‘the problem with the way things are commissioned is it’s commissioned competitively, so you’re fighting over the same pot of money’. Clinical teams whose expertise and experience ought to be shared and communicated are being separated and forced into damaging and unnecessary competition.

This model of competitive funding has also exacerbated precarity within sexual health medicine. ‘The commissioning is too short a time’, explained one medic, meaning that ‘the smaller organisations spend a lot of their time chasing the next funding stream, and you lose good staff’, whose jobs are not guaranteed because of short-term grants. With fewer clinicians entering sexual health medicine, and greater demand on existing staff to bid for funds, capacity to deliver services is dwarfed by demand. The clinicians had all experienced difficulties recruiting staff into sexual health posts. A genitourinary consultant reported the worrying, but by no means unique, fact that ‘we can’t staff our clinics’.

These experiences are causes for alarm, an alarm which a commissioner for sexual health services could raise. With a possible change in government on the horizon, professional bodies and representatives should lobby for such an appointment.
